# An agent-based approach for modelling and simulation of glycoprotein VI receptor diffusion, localisation and dimerisation in platelet lipid rafts

**DOI:** 10.1038/s41598-023-30884-6

**Published:** 2023-03-08

**Authors:** Chukiat Tantiwong, Joanne L. Dunster, Rachel Cavill, Michael G. Tomlinson, Christoph Wierling, Johan W. M. Heemskerk, Jonathan M. Gibbins

**Affiliations:** 1 School of Biological Sciences, University of Reading, Reading, UK; 2 Department of Data Science and Knowledge Engineering, Maastricht University, Maastricht, The Netherlands; 3 School of Biosciences, University of Birmingham, Birmingham, UK; 4 Alacris Theranostics GmbH, Berlin, Germany; 5 Department of Biochemistry, CARIM, Maastricht University, Maastricht, The Netherlands; 6 Synapse Research Institute, Maastricht, The Netherlands

**Keywords:** Computational models, Computer modelling

## Abstract

Receptor diffusion plays an essential role in cellular signalling via the plasma membrane microenvironment and receptor interactions, but the regulation is not well understood. To aid in understanding of the key determinants of receptor diffusion and signalling, we developed agent-based models (ABMs) to explore the extent of dimerisation of the platelet- and megakaryocyte-specific receptor for collagen glycoprotein VI (GPVI). This approach assessed the importance of glycolipid enriched raft-like domains within the plasma membrane that lower receptor diffusivity. Our model simulations demonstrated that GPVI dimers preferentially concentrate in confined domains and, if diffusivity within domains is decreased relative to outside of domains, dimerisation rates are increased. While an increased amount of confined domains resulted in further dimerisation, merging of domains, which may occur upon membrane rearrangements, was without effect. Modelling of the proportion of the cell membrane which constitutes lipid rafts indicated that dimerisation levels could not be explained by these alone. Crowding of receptors by other membrane proteins was also an important determinant of GPVI dimerisation. Together, these results demonstrate the value of ABM approaches in exploring the interactions on a cell surface, guiding the experimentation for new therapeutic avenues.

## Introduction

The plasma membrane of eukaryotic cells provides a physical and biochemical interface^1^ that allows the precise control of cell functions, facilitates shape change and movement^2^, attachment to the extracellular matrix or other cells, the controlled transfer of solutes outside-in and inside-out^3^, and the onset of the signalling mechanisms that regulate a cell^4^. Through the basic structure of its phospholipid bilayer, the plasma membrane provides a specialised environment in which cell surface receptors engage with extracellular ligands to trigger the transduction of signals in the cytosol. These signals are then propagated and amplified through enzyme cascades culminating in a controlled change in cell behaviour, for instance in gene expression, migration, secretion, proliferation, survival and apoptosis^5–7^.

Transmembrane receptors may move laterally within the phospholipid plane of the plasma membrane, although there are movement restraints due to the presence of and linkage to other surface proteins, as well as due to the presence of intracellular proteins, such as the membrane actin-myosin and tubular cytoskeletons^8,9^. The receptors may also be restricted in their movements due to the position of ligands, for instance, in the extracellular matrix^10^, or due to ligand-induced dimerisation or clustering, as in cases of the insulin and antibody receptors^11,12^. Interactions of plasma membrane receptors with other proteins inside the cell are furthermore controlled via biochemical processes such as post-translational modifications of proteins (phosphorylation, acetylation, ubiquitination, sumoylation, glycosylation, lipidation), ultimately leading to precisely regulated temporal and spatial control of cell signalling mechanisms^13–16^.

The ability of receptors to initiate cell signalling is influenced by the membrane phospholipid composition and distribution^7^. Small and transient nanodomains of the membrane enriched in cholesterol and glycolipids, known as lipid rafts, present unique physicochemical properties, enabling a highly localised enrichment of cholesterol and other lipid molecular species to influence membrane fluidity and the ability of proteins to move within^17^. The concept of intra-membrane heterogeneities and lipid rafts has thereby facilitated our understanding of the spatiotemporal orchestration of receptor signalling mechanisms.

Limited efforts have been made so far to develop theoretical models that combine the effects of intra-membrane constraints and ligand-induced actions with for understanding of the critical elements of receptor localisation and movement. One approach that can be used to study the dynamics of a particle on a membrane is agent-based modelling (ABM). Previous work has utilised this approach to investigate the formation of generalised molecular clusters^18^, finding that protein diffusion is influenced by its neighbourhood, or to investigate more specific questions about particular receptor classes (such as integrins^19^) without recourse to data. Das and coworkers^18^ have developed an in-house code to link data and agent-based models to answer specific questions centring on the activation of trafficking of EGFR-HER2 receptors.

**Figure 1:**
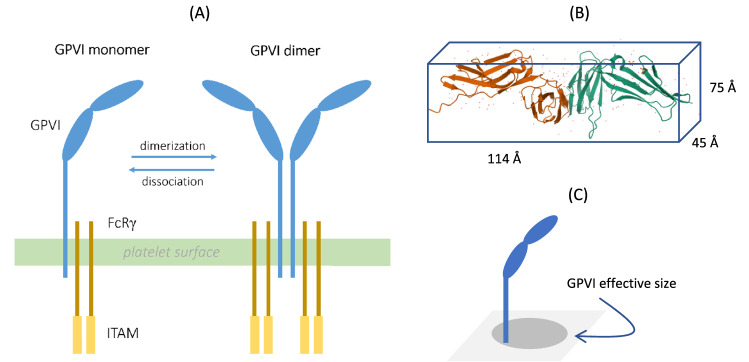
Structure and dimerisation of platelet GPVI receptors. (**A**) The extracellular domain of monomeric GPVI on platelets comprises of two IgG domains and a connection to the transmembrane domain (blue). The GPVI protein is stably connected to two chains of the FcR γ-chain, forming ITAM-containing signalling domains. Monomers of GPVI can dimerise with other monomers (dimerisation), a process that is reversible (dissociation). Adapted from Induruwa et al. (2016)^27^. (**B**) Crystal structure of human platelet GPVI. Image taken from RCSB PDB (rcsb.org), annotation PDB ID 2GI723. (**C**) Projected illustration of GPVI as a transmembrane protein with an assigned effective area in two dimensions.

For this study we constructed a simple and effective model, based on experimental evidence , for predicting receptor movements on the anucleate platelets using the ABM approach. Our chosen target was the receptor for collagen, glycoprotein (GP) VI, which is uniquely expressed on blood platelets and megakaryocytes^20,21^. The binding of collagen to this receptor leads to GPVI dimerisation and clustering, and to a signalling response that culminates in rapid thrombus formation, which contributes to haemostasis^22^. The monomeric GPVI receptor has a weak affinity for collagen and is non-covalently associated with the Fc receptor γ-chain, through which it transmits signals^23^. Receptor dimerisation results in the formation of a complex with a higher affinity for collagen (Fig. 1A), thus facilitating ligand binding and signalling responses^24–26^.

Developing an ABM with distinct regions of membrane lipid composition—here referred to as confined domains that are proxy entities for lipid rafts^28,29^—we studied how GPVI receptors on the platelet plasma membrane can switch between monomer and dimeric entities. Our modelling studies support the preferential enrichment for GPVI in lipid rafts, in agreement with experimental observations^30^. Through simulation of multiple facets of the plasma membrane and membrane proteins, we thus provide a basis for understanding how receptor complexes form and function, and can impact altered receptor signalling processes in disease.

## Methodology

### Application of agent-based modelling (ABM)

**Figure 2:**
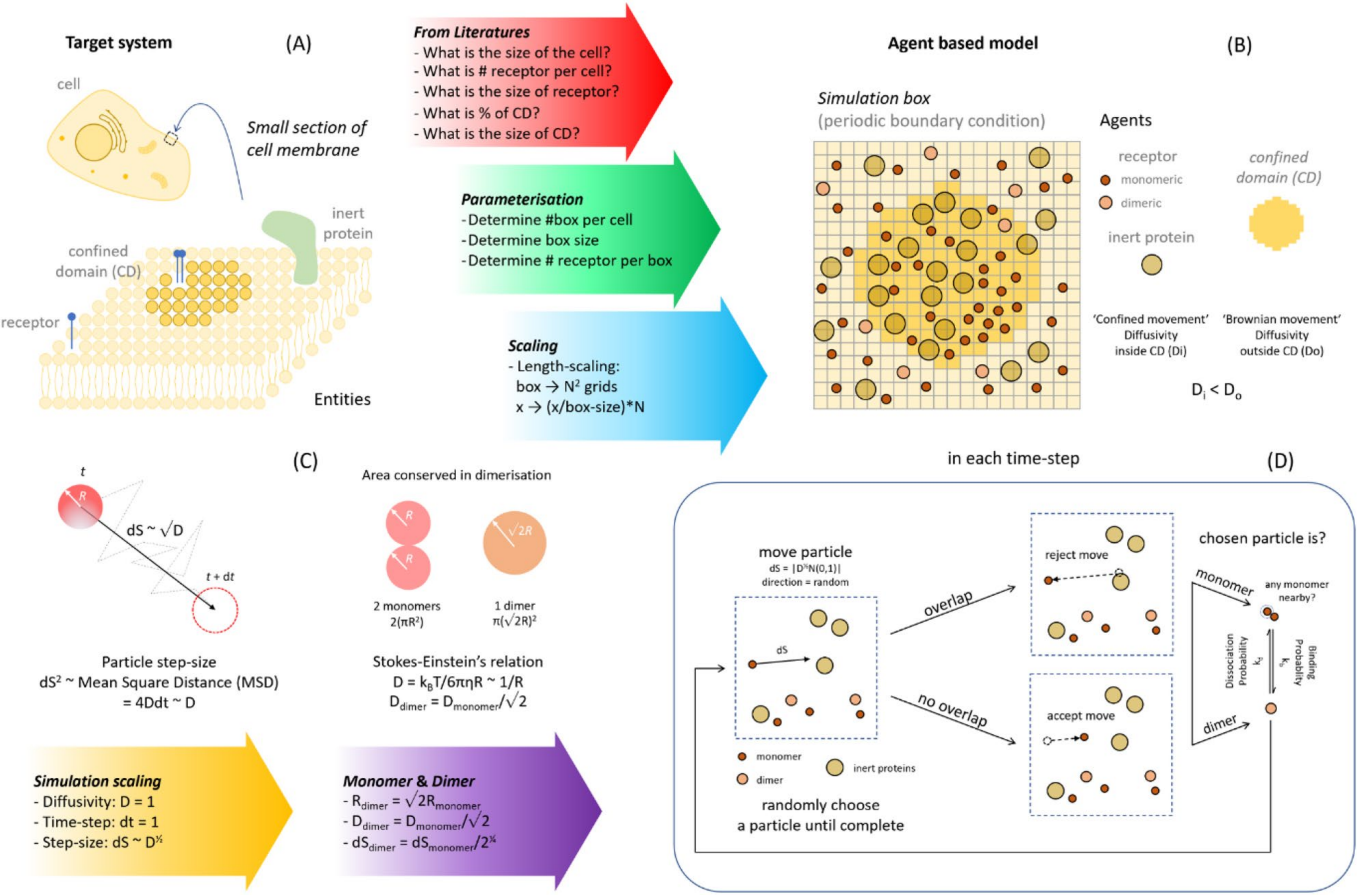
Overview of ABM simulation procedure. (**A**) The target system, i.e. the platelet membrane. The simplified version of a membrane consists of two areas, i.e. parts where molecules are confined in movements (confined domains), and the remaining part where they move freely (Brownian motion). In addition to inert proteins, the receptors of interest are indicated as transmembrane proteins. (**B**) Application of ABM to target receptor dimerisation. The membrane in the simulation box consists of agents (receptor molecules) in monomeric or dimeric forms and inert proteins. The confined domains are considered to represent lipid rafts. All agents are treated as independent, of which mathematical rules determine their properties and interactions. (**C**) Assignment of agent parameters. The simulation parameters included diffusivity, particle size and step size. (**D**) Rules for agent movements. Each simulation step consists of a randomly placed agent with random walk (rejected in case of overlapping), dimerisation and dissociation. Steps are repeated until all agents are selected, after which movements follow.

An ABM approach was used to simulate agents (receptors and lipids) on the cell surface^31^. This approach has been used in different fields of physical science, biological science, social science, and finances^32^. For example, several recently published works used ABM to study the spreading of the COVID-19 pandemic^33–35^. There are several ways to implement ABM, either by coding the model from scratch or using existing software. A commonly used ABM software package is NetLogo, which is multi-purpose, computationally efficient and easy to use, offering the advantage of being easily implemented and modified by non-theoretical experimentalists^36^. Using NetLogo, we simulated the diffusion of receptors in a two-dimensional plasma membrane. The implementation of this is demonstrated in Fig. 2A-D, and a flowchart is provided in the Supplement. The generated models can be easily modified to model different kinds of receptors and transmembrane proteins, by adjusting properties such as size, mass and diffusivity. To ease this modification, the code to run simulations is made available, and details on how to install and implement it are given in the Supplement. In our ABM approach, receptors are able to move with an assigned behaviour, which is either deterministic or stochastic as modelled. Certain areas of the plasma membrane were considered as confined areas with reduced diffusivity. By default, components in the system were studied in a two-dimensional box with periodic boundary conditions to imitate an infinite membrane^37^.

### Brownian motion

Agents (receptor molecules and other membrane proteins) were considered to move freely in the two-dimensional surface in random directions. By applying a mean square distance (MSD) of Brownian motion on a two-dimensional surface as time (*t*) dependent^38^:

$$ MSD = 4Dt, $$

a given step size (*dS* ~ *MSD*^½^) was taken, depending on the agent’s diffusivity (*D*) as *dS* ~ *D*^½^. Herein, the constant of variation was a function of the applied scaling. Agents in the simulation were modelled as circular discs, which never overlapped. It was assumed that the area occupied by one receptor is conserved during dimerisation, and that the space occupied by two monomers is equal to that occupied by one dimer, π*R*^2^_dimer_ = 2π*R*^2^_monomer_. The sizes (radii) of dimer *R*_dimer_ and monomer were then related as *R*_dimer_ = √2 *R*_monomer_ (Fig. 2C). The relationship of diffusivity and particle size was retrieved from the Stokes–Einstein relation^39^:

$$ R = {\text{k}}_{{\text{B}}} {\text{T}}/6\pi \eta D\sim1/D, $$

where k_B_, T, and η are Boltzmann constant, temperature, and viscosity, respectively. Although this formula is modelled in a 3-dimensional case, we presumed that the inversely proportional relationship between *R* and *D* was retained in 2-dimensions, the coefficient being absorbed in the scaling process. Combining these assumptions, the relationship between step size of monomer and dimer was:

$$ dS_{{{\text{monomer}}}} = 2^{1/4} dS_{{{\text{dimer}}}} $$

Note that the step size of an agent (receptor) in each time step may not be equal. In the calculation above, the maximum step size was set, but the actual step size in each movement could be generated according to a Wiener’s process, *dS*_actual_ ~|*N*(0,1)|*dS*_maximum_. Herein *N*(0,1) forms a random variable with a standard normal distribution (Fig. 2D).

Experimentally, using single-particle tracking, it has been seen that the diffusivity of GPVI molecules on mouse platelets decreased by approximately ten times, when present in regions with confined membrane properties^40^, with the receptor’s mode of motion changing from Brownian movement to restricted movement. In our ABM implementation, the mode of motion of the receptor inside and outside the confined domain remained the same; the only difference being the diffusivity. While the presence of this domain confined the movement of the receptor, we assumed that the receptor was effectively moved slower, with a smaller diffusivity within the domain.

### Receptor dimerisation

The effects of dimerisation and dissociation of receptors were captured by the probabilities *k*_*b*_ and *k*_*d,*_, respectively. Herein, dimerisation was defined as the conversion from two monomers to one dimer. The threshold of conversion was arbitrarily set at 10% of the monomer’s diameter. For calculating the conversion, a random number *R*_[0,1]_ ∈ [0,1] was generated. Dimerisation occurred if this number met the condition of *R*_[0,1]_ < *k*_*b*_. Conversely, dissociation was imputed as the change from one dimer to two monomers. For dimer movements, also a random number *R*_[0,1]_ was generated, and dissociation occurred when *R*_[0,1]_ < *k*_*d*_ (Fig. 2D).

### Parameterisation and scaling analysis

The following section explains how values were assigned to parameters. Note that when precise values for parameters were not available, order of magnitude estimates needed to be made, applicable to the platelet surface and the collagen receptor GPVI. The simulation conversion parameters estimated in the following session are summarised in Table 1.

**Table 1:** List of real-world and simulation parameters. See estimates in “Methodology” section.

Parameter	Real-world scale	Simulation scale
Size of simulation box	3.0 × 10^–7^ m	30
GPVI effective size	114 Å	3.8% of 30 ~ 1.14
GPVI diffusivity	0.091 × 10^–12^ m^2^/s	1
Expected step size of GPVI in a time-step	12.5 nm	(π/2)^½^ ~ 1.25
Time-step	0.43 ms	1

#### Platelet surface area

The platelet volume based on previous work^41^ was taken to be *V*_p_ ≈ 7.4 × 10^–18^ m^3^, allowing us to determine (by assuming that platelets are perfect spheres) the radius *R* and the surface area *A*:

$$ R \approx 1.2 \times 10^{{{-}6}} \;{\text{m}}, $$

$$ A \approx 1.8 \times 10^{{{-}11}} \;{\text{m}}^{2} . $$

Some assumptions needed to be made in considering the shape and volume of platelets, since their activation results in changes in morphology and membrane organisations. We reasoned that with the open canicular system exposed, following activation, the morphology of a platelet is closer to a sphere than a discoid. If an average discoid platelet is considered to have an average diameter of ~ 3 μm, the thickness of the cell can be determined as ~ 1 μm. Thus, the surface area of a platelet would be ~ 2.4 × 10^–11^ m^2^ (~ 33% more than a spherical shape). If we account for the contribution of the open canicular system (estimated to be ~ 25% of the plasma membrane surface)^42^, the total surface area will increase to 3.2 × 10^–11^ m^2^. However, since the open canalicular system is continuous with the plasma membrane, we assumed that the volume of a platelet remains constant during shape change. The consequences of a different receptor surface density is addressed in section 9 of the results. For the remaining simulations, we maintained a platelet surface area of 1.8 × 10^–11^ m^2^, consistent with spherical shape with a diameter of 2.4 μm.

#### Simulation box size

The (transient) confined domain diameter for a lipid raft of *d* ≈ 100 – 300 nm was obtained from an earlier publication^28^. For convenience, we used a raft size of 200 nm. Note that the size did not affect the model outcomes (see “Results” section). A model limitation is the assumption of the confined domain as a single circular area in the centre of a periodic box, implying that a too-small box can result in simulation artefacts. In other words, if a raft size is smaller than 30 nm, less than one receptor molecule will be present inside a box. Too-small number of receptors per box could also lead to high fluctuations in the simulation results. We further assumed lipid rafts occupy about 35% of the plasma membrane surface area^43^. The total area occupied by lipid rafts was then calculated as:

$$ A_{{{\text{raft}}}} \approx 35\% \times A \approx 6.4 \times 10^{{{-}12}} \;{\text{m}}^{2} . $$

Considering this as the total area of confined domains, with d ≈ 200 nm, the count of domains was:

$$ {\text{box}}_{{\text{per platelet}}} = A_{{{\text{raft}}}} /\left( {\pi \left( {d/2} \right)^{2} } \right) \approx 205. $$

In the simulation, the box area (consisting of one confined domain per box) was:

$$ A_{{{\text{box}}}} = A/box_{{\text{per platelet}}} \approx 9.0 \times 10^{{{-}14}} \;{\text{m}}^{2} , $$

with a box length of:

$$ L = \left( {A_{{{\text{box}}}} } \right)^{1/2} \approx 3.0 \times 10^{{{-}7}} \;{\text{m}}. $$

#### Receptor count per box

The number of GPVI molecules in a single platelet was estimated as ≈ 9600 copies^44^. This gave as a number of GPVI monomers per simulation box:

$$ {\text{GPVI}}_{{\text{per box}}} = {\text{GPVI}}_{{\text{per platelet}}} /{\text{box}}_{{\text{per platelet}}} \approx 9600/117 \approx 47 $$

#### GPVI receptor molecule dimensions

The molecular dimensions of GPVI were taken from its crystal structure^23^: 114 Å × 45 Å × 75 Å. Considering the extremum case that its longest side is the projected diameter of the GPVI on the platelet surface (Fig. 1B,C), we choose a d_GPVI_ ≈ 114 Å. The size of a GPVI monomer scaled to the box size then was:

$$ d_{{{\text{GPVI}}}}^{{{\text{scaled}}}} = d_{{{\text{GPVI}}}} /L \approx (114 \times 10^{{{-}10}} )/(3.0 \times 10^{{{-}7}} ) \approx 3.8\% . $$

#### Step size and time scale of modelling

The diffusivity of a single GPVI molecule in the membrane has been measured before^40^, *D*_exp_ ≈ 0.091 × 10^–12^ m^2^ s^–1^. From the mean square distance of particle on a two-dimensional surface moving in Brownian motion, the step size can be scaled as:

$$ dS^{2} \approx 4Ddt. $$

According to this equation, we could either pick dS and determine the scale of *dt* from dS or vice versa. To simplify the simulation, we scaled the step size to order O(1) by setting *D* ~ 1, *dt* = 1, and *dS* = *D*^½^|*N*(0,1)|. Note that the constant 4 was absorbed in the D scaling and that random Brownian motion was assumed to have a random standard normal distribution, N(0,1). With these definitions, the expected step size was calculated at:

$$ dS_{{{\text{e}} {\text{xpected}}}} \approx \left( {\pi /2} \right)^{1/2} \times \left( {L/30} \right) \approx 12.5 \times 10^{{{-}9}} \;{\text{m}}, $$

where *r*_expected_ = (π/2)^½^ is the expected distance determined by a standard normal distribution function, and 30 comes from the defined scaled box size. Hence, the time scale was set as:

$$ dt \approx dS^{2} /4D \approx \left( {12.5 \times 10^{{{-}9}} } \right)^{2} /\left( {4 \times 0.091 \times 10^{{{-}12}} } \right) \approx 0.43\;{\text{ms}}. $$

This time scale was small enough to capture the confined behaviour of particles, which occurs in seconds^28^.

#### Inert proteins

The modelling further included an unknown number of transmembrane proteins that have no interaction with the receptor of interest. The effect of a collision between proteins was already incorporated in the diffusion simulation via Brownian motion. The motion direction and step size changed randomly due to random encounters, implying that the presence of inert proteins was included by default. Additional parameters such as additional inert proteins (in arbitrary numbers) were used to check for effects on receptor dimerisation.

### Standard setup of ABM simulations

Simulations were performed in NetLogo 6.2.2 (Supplementary Figure S1), using the algorithm illustrated in Fig. 2 (for details see Supplementary Figure S2). A list of simulation parameters per research question is provided in Table 2. The default start setting was 47 receptor monomers that were uniformly distributed in a box representing the plasma membrane. Of note, this default did not take into account the heterogeneities caused by membrane cytoskeletal connections and receptor complexes, although the model may reach a non-uniform equilibrium after the simulation. Based on the calculations above, the diameter of GPVI was approximated as 3.8% of the length of the simulation box (scaled as 30 × 30 pixels). The movement speed of monomers was set to D^½^. Each simulation was run for ≥ 200,000 steps to ensure equilibrium. An average of the last 50,000 steps was used for the analysis.

**Table 2:** List of parameters used in each simulation. CD, confined domain. Please note in this context, k_d_ and k_b_ are implemented as a rate in unit time as described in the “Methodology” section.

Simulation type	Binding rate (k_b_) (per molecule per unit time)	Dissociation rate (k_d_) (per unit time)	Diffusivity outside CD (D_o_) (unit lenght^2^ per unit time)	Diffusivity inside CD (D_i_) (unit lenght^2^ per unit time)	CD occupied area (%)	Number of added inert proteins (molecule)	Number of inert protein packs (dimensionless)	Fold number of CD merging (dimensionless)	GPVI number per platelet (% of 9600)
1. Receptors in CD area vs D_i_	0	0	1	1,2^−1^…,2^−9^,2^−10^	35	0	0	1	100
2. Dimerisation (with CD) vs D_i_	0.05	0.01	1	1,2^−1^…,2^−9^,2^−10^	35	0	0	1	100
3. Dimerisation vs %CD & D_i_	0.05	0.01	1	1,2^−1^…,2^−4^,2^−5^	0,5…,75,80	0	0	1	100
4. Dimerisation vs CD merging	0.05	0.01	1	0.1	35	0	0	0.5,1…,7.5,8	100
5. Added inert proteins	0.05	0.01	1	1	0	0,25…,175,200	0,25…,175,200	1	100
6. Disintegrated inert proteins	0.05	0.01	1	1	0	100	1,2^1^…,2^7^,2^8^	1	100
7. Dimerisation (w/o CD) vs D	0.05	0.01	2^−5^,2^−4^…,2^4^,2^5^	1	0	0	0	1	100
8. Receptors in CD area vs %CD	0, 0.01, 0.005, …, 0.000625	k_b_/5	1	0.1	15,16,…,22	0	0	1	100
9. Dimerisation vs receptor number	0.05	0.01	1	0.1	35	0	0	1	25, 50, …, 150, 175, 200
10. Dimerisation vs k_b_ & k_d_	60, 70, …, 130, 140% of 0.05	60, 70, …, 130, 140% of 0.01	1	0.1	35	0	0	1	100

Except where indicated otherwise, binding and dissociating probabilities were arbitrarily defined as *k*_*b*_ = 0.05 molecule^–1^ per unit time and *k*_*d*_ = 0.01 per unit time (or per timestep, dt ~ 0.43 ms as calculated above). These numbers were chosen to ensure balancing of the time scale of dimerisation and dissociation, i.e., to prevent an equilibrium without dimers or monomers. This also ensured that the number of GPVI molecules in dimeric form in the simulations were broadly consistent with the dimeric levels measured experimentally^26^. The impact of variation of these parameters is shown in the results (see “Simulation of ligand binding increases GPVI dimerisation”). All simulations were repeated three times. The code for this model, together with the setup for each simulation, is available in the Supplement.

## Results

In the present study, we aimed to understand how a fluid-mosaic plasma membrane influences receptor diffusion, interaction or dimerisation, and the initiation of cell signalling. According to the mosaic model, the phospholipids and proteins are not uniformly distributed. Lipid patches (rafts) are considered to concentrate signalling proteins, including receptors, thereby permitting or enhancing cell signalling processes^17,30^. Precisely how this occurs has not yet been resolved. The agent-based modelling (ABM) approach allowed us to explore the impact of confined lipid domains within the plasma membrane on the enrichment and clustering of the collagen receptor GPVI. The model can easily be applied to other receptors and cell types of interest, with adapted parameters as in the methods section.

To address ABM simulations, we designed a receptor-containing simulation box, representing a defined square part of the plasma membrane with mobile GPVI molecules and initially a single confined domain (“raft”). With the chosen parameters, we assumed that GPVI monomers have no inherent tendency to form dimers or clusters.

### Simulated receptors preferentially localise to confined domain areas

Differential diffusivity in the lipid domains of a membrane may result in an uneven distribution of transmembrane proteins. In the present ABM, we assumed that the confined domains contain a higher level of proteins that are free to move inside or outside^45,46^. In a series of simulations, we tested this idea.

The proportion occupied by the confined domain, as assumed in rafts, was estimated as 35% by Prior et al.^39^. The size of the confined domain was fixed as a circle, which represented a domain of lower protein diffusion. The diffusivity ratio outside and inside the confined domain was varied to simulate effects on receptor diffusion.

**Figure 3:**
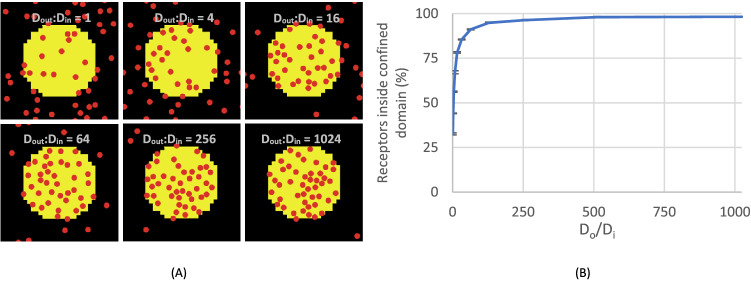
Preferential localisation of single receptors in the confined domain. (**A**) Snapshots of 11 simulations of 47 receptors (red dots) moving on the simulated membrane with confined domain (yellow circle). Note the sub-micrometer size of the simulation box of 0.3 × 0.3 μm, and the initial random distribution of GPVI receptors. Simulations were run for ≥ 200,000 steps, with an average of the last 50,000 steps shown. The diffusivity ratio between outside and inside confined domains, D_out_:D_in_, was changed from 1 (2^0^) to 1024 (2^10^). The snapshots shown are for D_out_:D_in_ = 1, 4, 16, 64, 256 and 1024. (**B**) Effect of an altered ratio D_out_:D_in_ on number of receptors inside the confined domain. Each simulation was repeated three times, means ± SD.

If the receptor localisation is not affected by diffusivity, the relative numbers of receptors located inside or outside the confined domain will be similar for all diffusivity ratios. In Fig. 3A, the ratio of diffusivity of receptors between the outside and inside of a confined domain, expressed as D_out_:D_in_, was taken as an independent variable and then changed from 2^0^, 2^1^, 2^2^, … to 2^10^ (i.e., 1024). The actual ratio can be estimated to be ~ 10, according to single particle tracking results of GPVI molecules in mouse platelets^40^. In our studies we varied this ratio from 1 to 1024 to explore the extreme relationships between diffusivity and location preference of GPVI. The number of receptors located inside the domain, as a dependent variable, was found to asymptotically reach 100%, with 50% at a D_out_:D_in_ of in the range of 8 to 16 (Fig. 3B). Note that if a different diffusivity ratio does not affect the receptor localisation, this number should not deviate from the starting value of 35%. Based on the obtained changes at default model settings, we concluded that GPVI receptors will preferentially localise to the confined domains, i.e., the areas with lower diffusivity.

Previous studies have demonstrated that GPVI is present in cholesterol-rich lipid rafts. GPVI recruitment occurs upon platelet adhesion to collagen^47^, a process which can lead to GPVI clustering^48^. While these membrane structures concentrate specific signalling proteins within, recent studies reveal that lipid rafts also cage or restrict protein and receptors diffusion^49,50^, which may be a prerequisite for GPVI clustering. Indeed, a heterotypic interaction of GPVI with PECAM1 is increased in lipid rafts^51^. Considering that lipid rafts can orchestrate the GPVI signalling^52^, we hypothesized that lowered diffusivity in rafts compared to non-raft domains results in an increased GPVI dimerisation within.

### Decreasing diffusivity in the confined domain increases receptor dimerisation

We then explored how the confined domain affected the likeliness of receptor dimerisation, a process that is known to enhance GPVI ligand-binding properties^24–26^. For simplicity in the ABM simulation, we assumed that dimerisation is not modulated by other proteins in the plasma membrane or actin cytoskeleton. We thus assumed that the fraction of receptors in dimeric form remains the same for all D_out_:D_in_ ratios.

**Figure 4:**
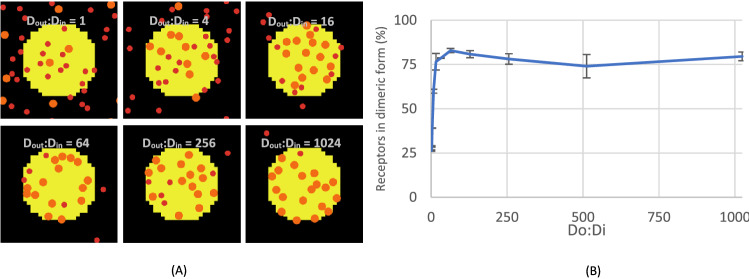
Higher diffusivity ratio enhances receptor confinement and dimerisation. (**A**) Snapshots of simulation of receptors in monomeric (red) or dimeric (orange) forms in the presence of a confined domain (yellow circle). Initially, 47 monomeric receptors were randomly distributed without dimeric form. Simulations were run for ≥ 200,000 steps, with an average of the last 50,000 steps shown. The diffusivity ratio between outside and inside confined domains, D_out_:D_in_, was varied from 1 (2^0^) to 1024 (2^10^). Snapshots are shown for D_out_:D_in_ = 1, 4, 16, 64, 256 and 1024. (**B**) Effect of altering the ratio of D_out_:D_in_ on the number of receptors in dimeric form. Simulations were repeated three times, means ± SD.

As illustrated in simulation snapshots (Fig. 4A), we found that an increase in the diffusivity ratio (i.e., lower diffusivity in the confined domain with D_out_:D_in_ set from 2^0^ to 2^10^) yielded a higher number of receptor dimers. Herein, the ratio of diffusivity of receptors outside or inside the confined domains was taken as an independent variable. The dimeric receptors increased non-linearly with the diffusivity ratio to reach a saturation level of 80% (Fig. 4B). The simulation thus pointed to a main effect of intra-membrane differences in receptor diffusivity for promoting receptor dimerisation.

### Total area of the confined domain influences receptor dimerisation

**Figure 5:**
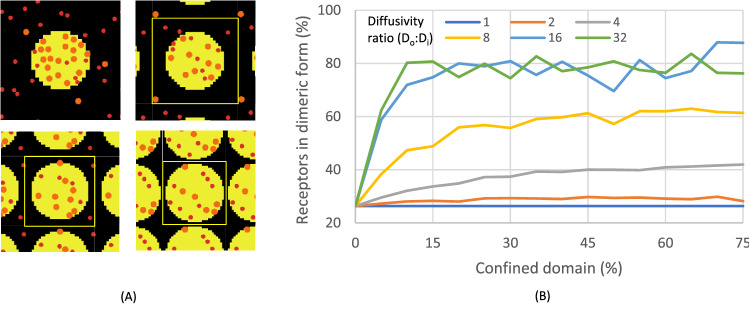
Increasing confined domain area induces more receptor dimerisation. Effect of increasing the confined domain area at different diffusivity ratios. (**A**) Snapshots of the occupied area of the confined domain (yellow circle) from upper left at 20%, 40%, 60% and 75%. Red and orange dots represent monomeric and dimeric receptors, respectively. Note that at higher area percentages, the number of receptors per box reduces, when the number of boxes per cell increases. The size of the confined domain was kept constant. (**B**) Results of simulation for receptor fractions in dimeric form. Simulations were run for ≥ 200,000 steps, with an average of the last 50,000 steps shown.

To explore whether the relative size of a confined domain affected dimerisation, this domain was again set as a circular area, of which the relative radius was altered to make up an increasing part of the membrane box size (Fig. 5A). The area occupied by the confined domain was then modelled from 0–75%, i.e., up to twice the estimated area of lipid rafts, while the diffusivity ratio D_out_:D_in_ was varied from 1 to 32. We found that both the area occupied by confined domains and the diffusivity ratio greatly affected the average number of dimers. Interestingly, the number of receptor dimers increased substantially from 10% to plateau to 40%, when the D_out_:D_in_ increased (Fig. 5B). The highest dimer levels were reached at the two highest D_out_:D_in_ ratios of 16 and 32. In addition, a larger area occupied by the confined domains was needed to plateau at lower D_out_:D_in_ ratios. In other words, the level of dimerisation increased with the diffusivity ratio, with curves reaching a saturation point at the lower domain area in case of a higher diffusivity ratio. Translated to receptor biology, this suggested that both the attraction strength and the size of raft-like structures can determine the extent of receptor dimerisation.

### Merging of confined domains does not influence receptor dimerisation

**Figure 6:**
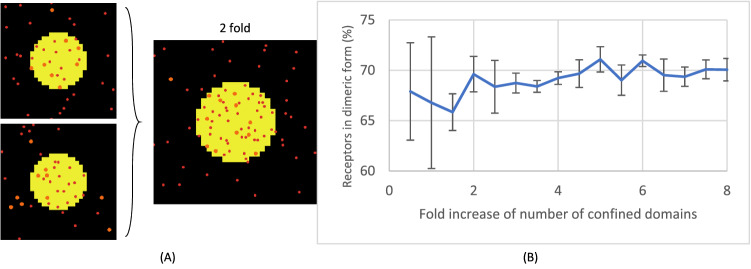
Merging of confined domains has no effect on receptor dimerisation. Simulated was the effect of merging two confined domains while fixing the total occupied area size. (**A**) Snapshots of two confined domains merged into one (yellow circles). The red and orange dots represent monomeric and dimeric receptors, respectively. (**B**) Simulation for determining dimeric receptors as a function of the fold merging of confined domains. Simulations were repeated three times, mean ± SD; Pearson correlation of 0.40 indicates a weak positive correlation between confined domain folds and dimerisation.

Since membrane rafts are temporary structures that can reversibly merge^53^, we hypothesised that the merging could affect receptor dimerisation. To assess this, we varied the number of confined domains while fixing the total area occupied, and then simulated the receptor organisation. Herein, we set the ratio of outside/inside diffusivity of receptors D_out_:D_in_ to 10, knowing that about half of the GPVI receptors on mouse platelets have a diffusivity approximately ten times lower than the other half of receptors with Brownian motion^40^. The simulation is visualized by snapshots in Fig. 6A. When extending this domain number to higher fold merging, we observed no change in dimer formation (Fig. 6B). Translating to real life, for platelets this suggests that the mere merging of membrane rafts does not impact receptor dimerisation.

### Inert protein crowding in the membrane increases receptor dimerisation

**Figure 7:**
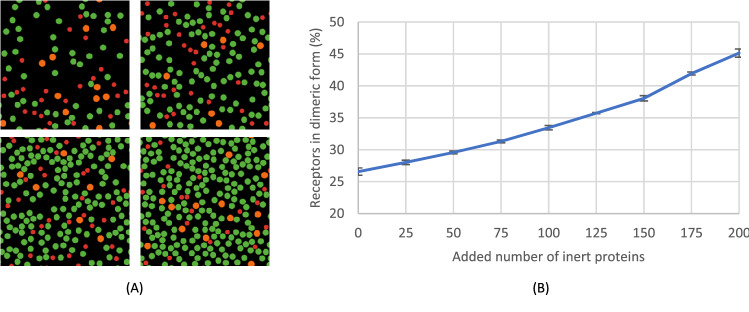
Increasing inert protein crowding induces more receptor dimerisation. Simulation of added inert proteins on the receptor dimerisation. Red and orange dot represents monomeric and dimeric receptors, respectively; green dots represent inert proteins. Simulations were run for ≥ 100,000 steps, with an average of the last 50,000 steps shown. (**A**) Snapshots from the left top with inert proteins of 50, 100, 150 and 200. (**B**) Plot of dimer counts versus number of inert proteins. Note the more abundant dimeric receptors, when protein crowdedness increases. Each simulation was repeated three times, mean ± SD.

As the platelet membrane contains other moving transmembrane proteins without interaction with the GPVI receptor, we also added free-moving membrane proteins to the ABM, acting as obstacles to receptor diffusion. In our simulation, the number of inert proteins per box varied from 0, 25, 50, … to 200 (Fig. 7A). The size of inert proteins was arbitrarily set at 0.05 of the box size, and their speed was set at 0.5D^½^. The average number of receptors in dimeric form, as an outcome variable, almost linearly increased from 25 to 45%, while the number of inert proteins increased from 0 to 200 (Fig. 7B). This is explained by the space occupied by the inert proteins, thus tightening the diffusion room of monomeric receptors, which then leads to a higher encounter rate between receptors.

### Disintegration of inert proteins has a minor impact on receptor dimerisation

**Figure 8:**
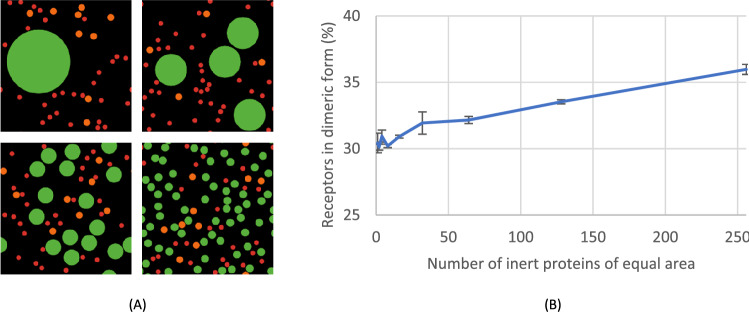
Disintegration of inert protein slightly affects receptor dimerisation. Simulation of the effect of size of inert proteins on receptor dimerisation. The total area occupied by inert proteins was kept constant, while subareas of smaller size were created. See further Fig. 5. (**A**) Snapshots for 1, 4, 16 and 64 splits of inert proteins. (**B**) Plot of receptor dimer counts versus the number of disintegrated inert proteins. Each simulation was repeated three times, mean ± SD (Pearson correlation + 0.96).

We then considered that inert proteins could differ upon platelet activation, i.e., the proteins can become aggregated or disintegrated^54^. This was simulated by splitting the space size into smaller components while not changing the total space occupied by inert proteins. Inert proteins were placed randomly in the simulation box, and the inert protein size was initially set as one large circle with a diameter half of the box size. Then the protein number was increased from 1 to 256 (2^0^ to 2^8^), while the size was proportionally decreased with a total conserved area (Fig. 8A). According to the Stokes–Einstein relation^39^, diffusivity may be expected to increase since smaller particles move faster. Yet, our ABM simulations showed a minor increase from 30 to 36% of dimeric receptors, when the inert protein disintegrated from 1 to 256 pieces (Fig. 8B). To verify that this was not statistical noise, we determined a Pearson correlation coefficient of + 0.94. Accordingly, it appears that the disintegration of inert proteins exhibits only a minimal effect on receptor dimerisation.

### Decreasing receptor diffusivity increases the level of receptor dimerisation

**Figure 9:**
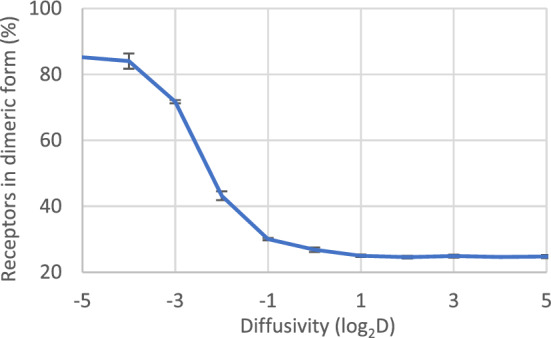
Decreasing receptor diffusivity increases dimerisation. Simulated testing of altered receptor diffusivity to assess receptor dimerisation, with otherwise fixed parameters. For convenience, receptor velocity was taken as v = D^½^. Diffusivity varied from 2^–5^, 2^–3^, …, to 2^5^. The simulation shows a decrease in dimer number at an increased diffusivity. Data are shown in a semi-log2 scale; mean ± SD (n = 3 simulations).

According to work by Haining et al.^40^, the activation of GPVI decreased in Tspan9 knock-out mice, while also the overall diffusivity of GPVI decreased. This suggested that a reduced diffusivity per se can lead to reduced dimer formation. To test this, we simulated the variation of receptor diffusivities from 2^–5^, 2^–6^, … to 2^5^; and then measured dimerisation, taken as a proxy for receptor activation. It appeared that the number of dimeric receptors, as a dependent variable decreased substantially from 85 to 25%, when the diffusivity increased (Fig. 9). In other words, a slower-moving agent has a higher chance of encountering other agents. This suggests that the phenotype of reduced GPVI signalling of Tspan9-knock-out platelet is unlikely to be explained by changes other than membrane diffusion alone.

### Estimation of the plasma membrane area of confined domains

Haining et al*.*^40^ deduced the proportion of the plasma membrane that constitutes confined domains using a single particle tracking microscopy, noting that GPVI exhibited distinctive Brownian and confined movement without and within confined domains, respectively. The number of GPVI molecules in Brownian and restricted movement mode was approximately equal^40^. A temporal change in the proportion of the membrane confined domains may also impact the localisation of a receptor. Using electron microscopy and spatial point pattern analysis, previously lipid rafts were estimated to comprise approximately 35% of the total membrane surface^43^.

**Figure 10:**
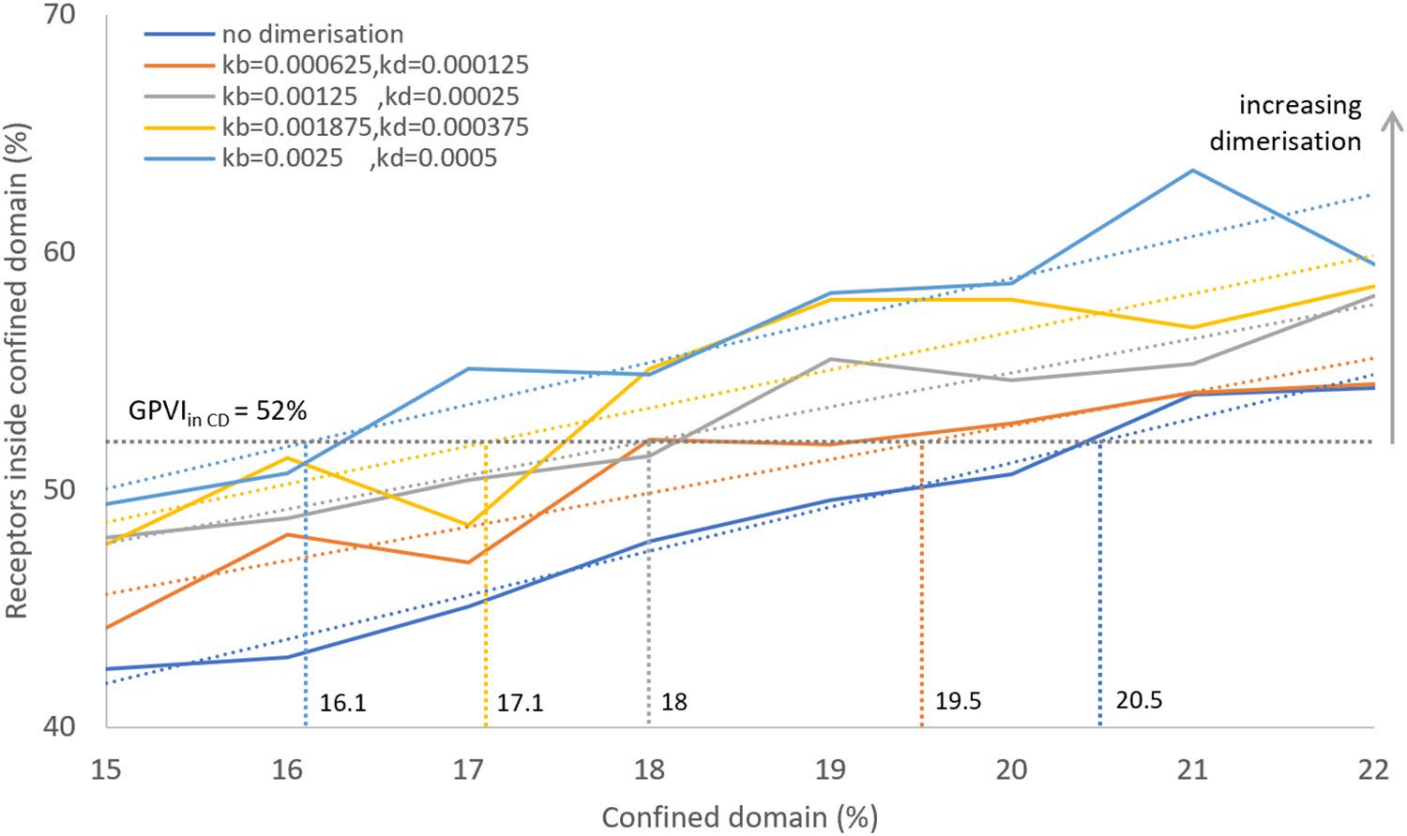
Enforced GPVI dimerisation reduces the confined domain area for a given GPVI localisation ratio. Plot of simulation of GPVI localisation in the presence of a confined domain with variable occupied area. Note the near linear increase of receptors inside the confined domain when this area increases. The reported value of GPVI with restricted movements^40^ is about 50%, pointing to a confined domain size of 20–21%. In the presence of dimerisation, this area slightly decreases to 19–20%, with a k_b_ = 0.000625 and k_d_ = 0.000125 (least square regression analysis).

We used ABM to ask what proportion range of the plasma membrane should comprise a confined domain, such in accordance with the 50% of GPVI receptors with restricted movements^40^. To answer this, we fixed the diffusivity ratio to D_out_:D_in_ = 10:1 and the size of the domain to 200 nm, and then varied the percentage of the plasma membrane occupied by a confined domain. The number 10:1 was obtained from the diffusion coefficients of two pools of GPVI^40^. A first run of the simulation gave 20–21% of confined domains, which is below the estimation of lipid rafts of 35%^43^. Subsequently, the effects of enforced dimerisation were added (Fig. 10). The adding of dimerisation somewhat decreased the corresponding domain area (with GPVI_inside_ ~ 50%), meaning that the area occupied by the confined domain would not exceed 21%, based on the model prediction. We therefore concluded that, while confined domains govern the receptor dimerisation rate, the physicochemical properties of these do not alone control receptor function. Other constraining features such as more complex receptor interactions, including the actin-based membrane skeleton within lipid rafts^55^ and receptor crowding, are also important.

### Increased receptor surface density results in higher predicted dimerisation levels

Several estimates may affect the number of receptors on the cell surface used in the current model. The first variable is the number of GPVI receptor copies. We set this number at 9,600 per platelet, following Burkhart’s work^44^, which was obtained by quantitative mass spectrometry. Other studies using flow cytometry reported different figures ranging from 3,000 to 9,000^56,57^, while also different *GP6* alleles lead to altered membrane-expressed GPVI levels^58^. Furthermore, even within a given subject, platelet sub-populations exist with > tenfold differences in GPVI level, related to ageing cells^59^, differential cell size, receptor internalisation and shedding^60^.

Parameter estimation in this model assumed the platelet to be a perfect sphere; in reality, the disc-like shape of platelet leads to a higher surface area given the same volume. Also human and mouse platelets differ in this respect. For mice, the GPVI density can be estimated as 575 molecules per μm^2^ (mouse platelet volume of ~ 4.7 fl^61^, with GPVI ~ 7800 molecules per platelet^62^). Considering that the dimerisation rate depends on receptor density, inter-species differences can also be captured by the current simulation.

**Figure 11:**
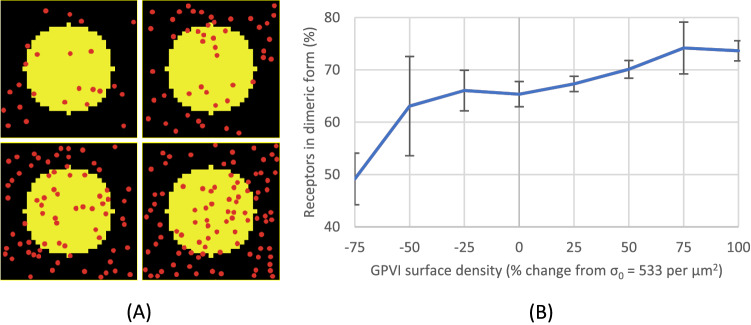
Increasing GPVI surface density increases dimerisation. (**A**) Simulation setups with various surface densities of GPVI: from left to right, top to bottom 50%, 100%, 150%, and 200% of σ_0_. (**B**) The plot shows that receptor dimerisation (as proportional to total GPVI, in %) increases with the GPVI surface density (as proportional to σ_0_ in %). Reference density σ_0_ was set as 533 molecules per μm^2^ (9600 receptors divided by platelet surface area ≈ 1.8 × 10^–11^ m^2^; spherical assumption).

While setting for human the GPVI density as 9,600 per platelet surface area ≈ 1.8 × 10^–11^ m^2^ (σ_0_ = 9600/1.8 × 10^–11^ m^2^ = 533 molecules per μm^2^) as a reference, we varied this density from −75 to + 100% from σ_0_ and measured the percentage of receptors in dimeric form (Fig. 11). The simulation predicted that an increased surface density of GPVI elevates the dimeric GPVI from 50 to 75% (over a range of −75 to + 100% of reference levels). This imply that in the model dimerisation does not increase proportionately with the ratio of density.

### Simulation of ligand binding increases GPVI dimerisation

**Figure 12:**
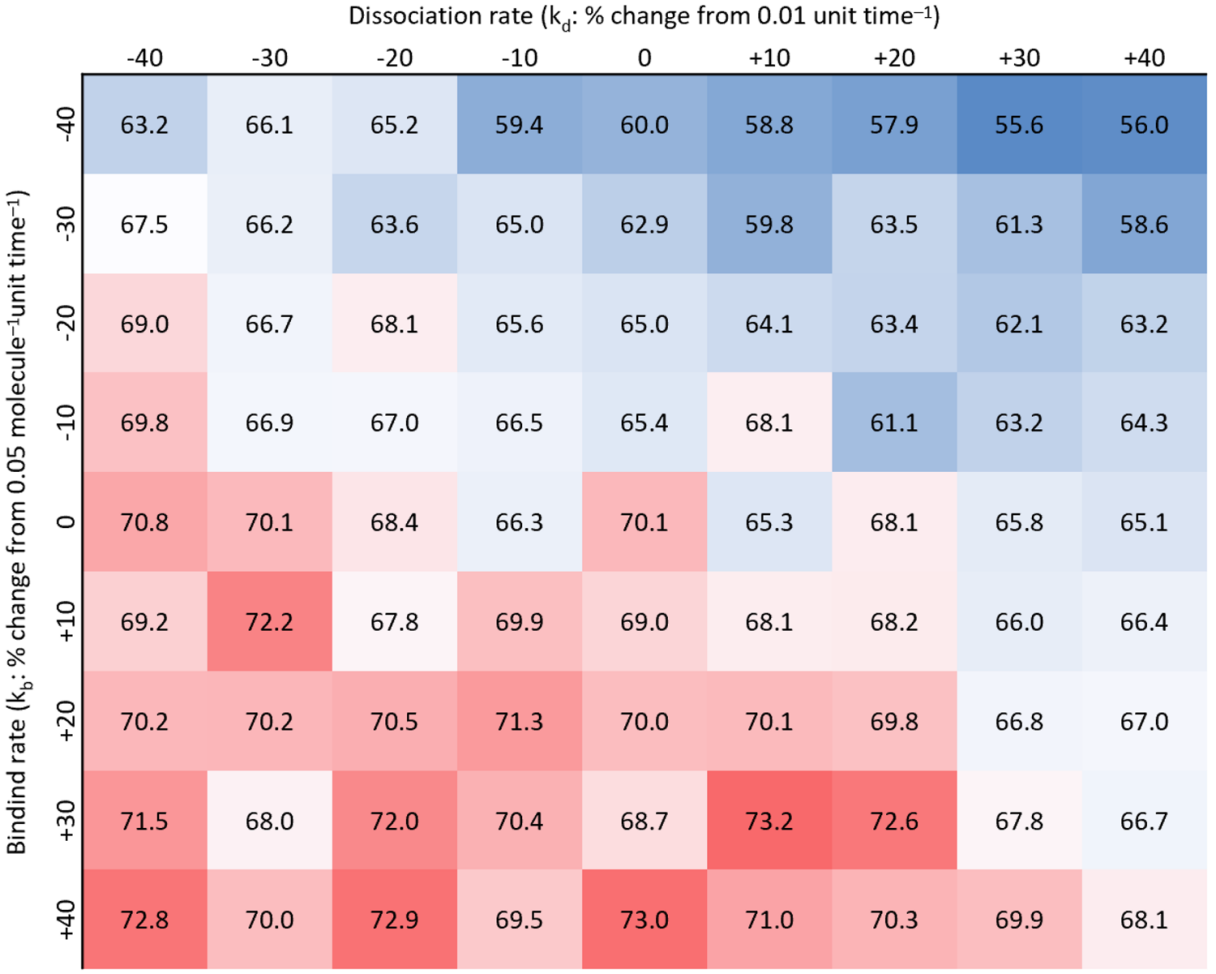
Increase in binding rate and decrease in dissociation rate increase GPVI dimerisation. Simulation varying the binding and dissociation rate value from −40%, −30%, …, + 30%, + 40%, deviating from the initial values of k_b_ = 0.05 molecule^–1^ unit time^–1^ and k_d_ = 0.01 unit time^–1^. Values represent percentages of GPVI in dimeric form, as a proportion of total GPVI. Red colour represents higher dimerisation, blue lower dimerisation.

A way to simulate the effect of ligand binding is to increase the GPVI binding rate and/or dissociation rate. In real life, we expect GPVI in dimeric form to increase and to remain dimeric on collagen-adhered platelets^26^. To simulate this, we varied the k_b_ and k_d_ from the initial values (k_b_ = 0.05 molecule^–1^ unit time^–1^ and k_d_ = 0.01 unit time^–1^) by ± 10%, ± 20%, ± 30%, and ± 40%. The percentage of receptors in dimeric form was then simulated, as displayed in Fig. 12. In this case, the dimerisation rate to increased when the binding rate was increased and/or dissociation rate decreased – both may illustrate the effect of ligand binding. A decrease in dissociation rate means that a formed dimer is more stable (e.g. stabilised by a multimeric ligand), while an increase in binding rate allows monomers to form into dimers with greater probability (induced by receptor-associated proteins).

## Concluding remarks

**Figure 13:**
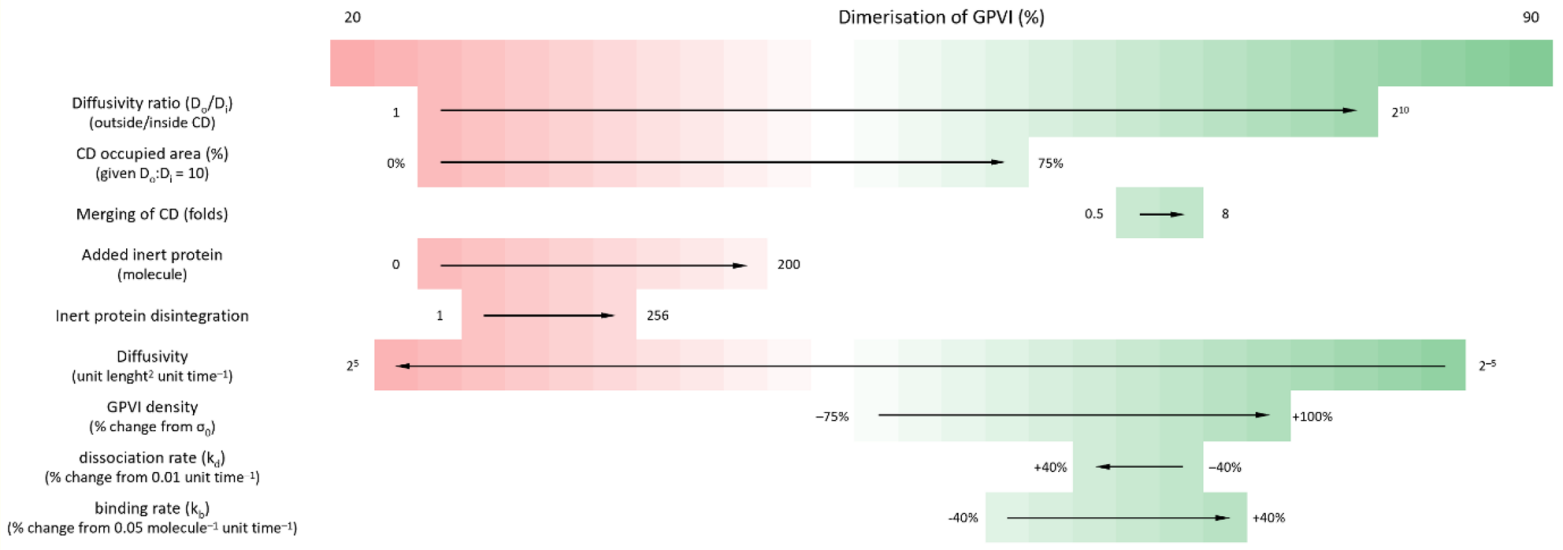
Relative contributions of various modelled factors to GPVI dimer formation. Arrows show the direction of increasing the indicated factor. Other conditions were assumed to be fixed, when varying the parameter of interest. The level of dimerisation (in %) displayed in colour scale: from 20% (red) to 90% (right).Numbers indicate parameter ranges tested with units or dimension indicated in parentheses.

In this study, we have demonstrated the abilities of a simple ABM technique to understand the constraints of receptor localisation and movement in the plasma membrane. Receptor dimerisation and subsequent clustering upon ligation are key initiators of signal transduction by many receptors that regulate cell function, including cell adhesion, migration and activation, for instance in the context of haemostasis and immunity. The ABM illustrates the presence of different lipid domains with distinctive properties (as confined domains), the space that these occupy on the cell surface, and the importance of the plethora of additional proteins on the cell surface, that form crowds and influence a given receptor’s ability to interact with partner proteins. The relative contributions of the functionally relevant parameters tested in the ABM to GPVI dimerisation levels are summarised in Fig. 13.

Due to its simplicity, computational efficiency and ease of use, ABM has the potential to be developed and generalised also to other cell types and more complex systems of receptor/protein or cell membranes. It can be applied to various studies by adapting the properties of agents (e.g., mass, size, environment), and how these affect agent diffusivity and interaction rules. Moreover, the present still simple ABM can be further developed into a more complex system with more agents and conditions. Useful additions such as receptor interactions with the cytoskeleton can be added in by utilising a computational cluster^63,64^.

Taken together, this study forms an initial step to model and define membrane properties and their influences on receptor function. This will highlight specific processes that may be targeted therapeutically to increase or decrease receptor function and may be used for teaching, enabling the impact of modulation of various model components to be tested or demonstrated in silico.

## Supplementary Information

Supplementary Information.

## Data Availability

All data generated or analysed during this study available from the corresponding author on reasonable request.
