# Supplementary material for: An agent-based approach for modelling and simulation of glycoprotein VI receptor diffusion, localisation and dimerisation in platelet lipid rafts
Source: Sci Rep. 2023 Mar 8;13:3906. doi: 10.1038/s41598-023-30884-6 (PMC9994409; doi:10.1038/s41598-023-30884-6)
Supplement: Supplementary file 1 — Supplementary Information. [file 41598_2023_30884_MOESM1_ESM.docx]

**Supplementary**

The program implemented in this work is uploaded to the following reference.

Tantiwong, Chukiat (2022): *ABM_for_transmembrane_receptor_diffusion_and_dimerisation.nlogo*. figshare. Software. <https://doi.org/10.6084/m9.figshare.20444046.v2>

In order to use the program, you need to install NetLogo. Please download NetLogo via

<https://ccl.northwestern.edu/netlogo/download.shtml>

Open the downloaded program with NetLogo; you should be able to see an interface, as shown in Figure S1. You can change the value of parameters, click on setup and run to visualise the movement of agents. The tick’s sliding tab can be used to speed up the simulation. The plot on the right-hand side can be exported with right-click. The info tab shows the description of how the model work, the definition of each parameter, things to notice, and things to try. The code tab contains raw code to run the model, with descriptive comments.


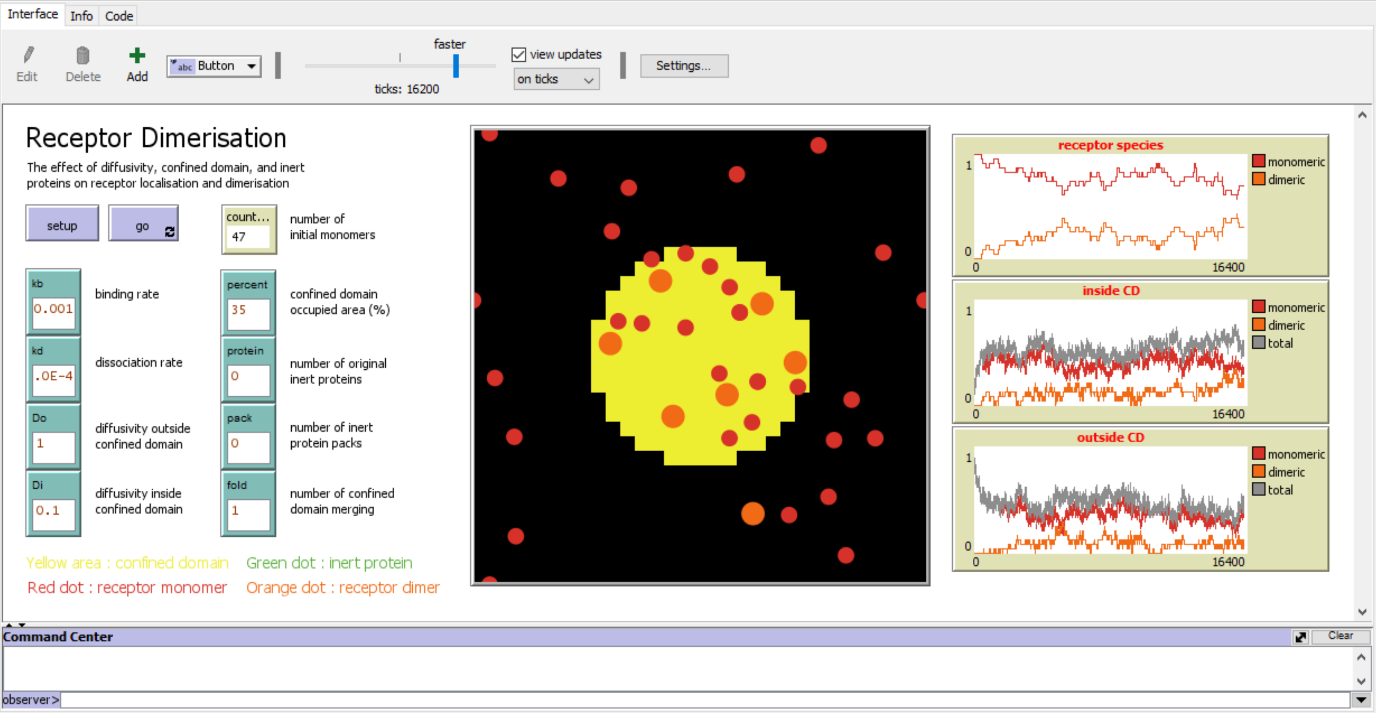


**Figure S1. NetLogo screenshot of applied ABM**. A screenshot was taken from the NetLogo program, and contains parts of interfaces, information, and codes. The shown interface part visualises the simulation box, the time-plots of receptor monomers and dimers, and the adjustable parameters. The information part describes the model and parameters. The coding part contains relevant details.


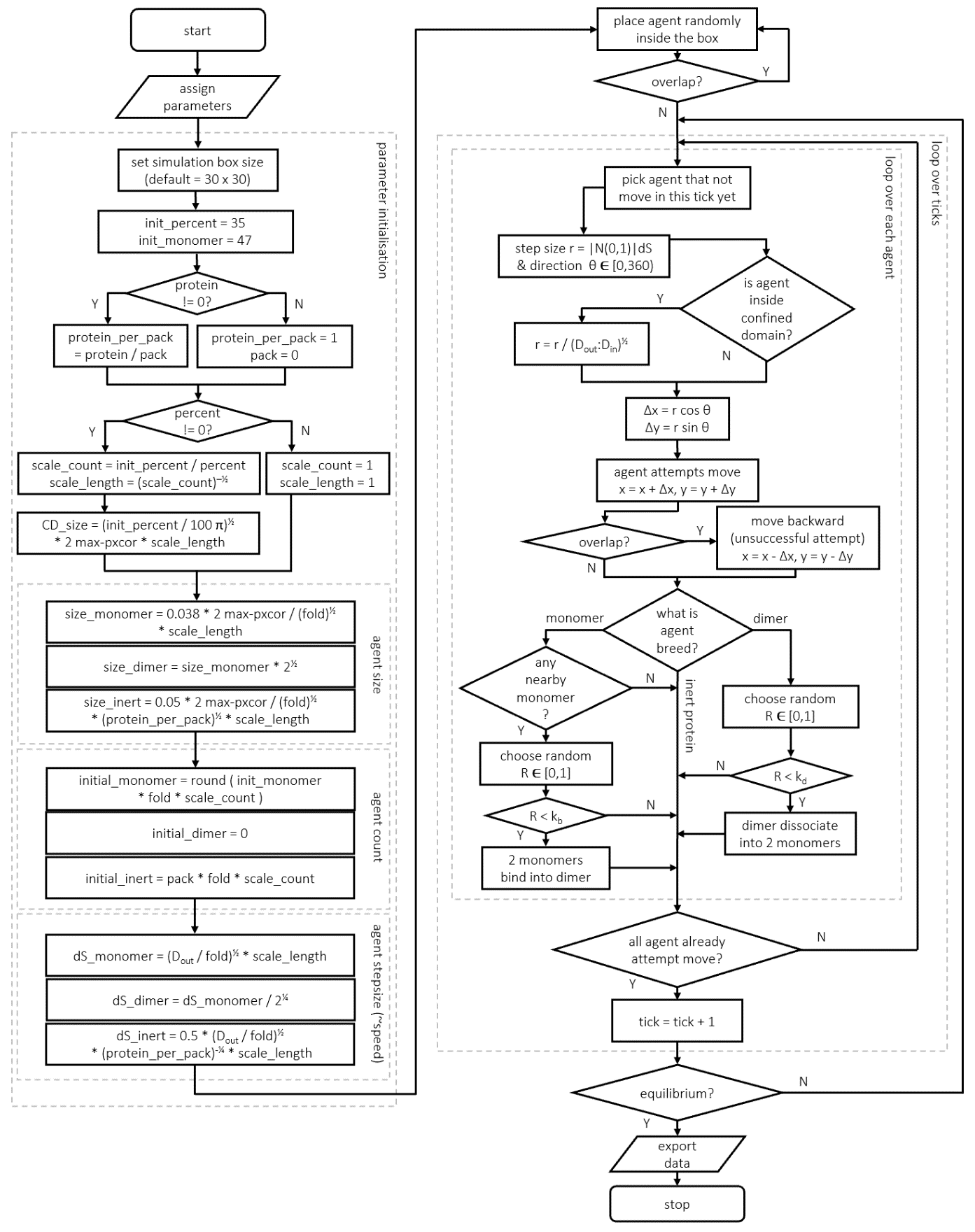


**Figure S2. Flowchart of algorithm used in the GPVI-receptor ABM**. The algorithm includes various steps in the simulation from randomly assigned initial positions up to stochastically movements per agent.
